# Indents

**Published:** 1916-09

**Authors:** 


### INDENTS

**☞ Brief Articles of Technical or Scientific Value will receive notice and comment. Contributions invited.**

*Reflex Irritations.* We undoubtedly have a large group of disorders that seem to have a very definite connection with physical irritations about the teeth. At the Miller Memorial meeting recently held in Columbus, while seated at the banquet table, a note was handed to me in which the writer stated that he wished to call my attention to the fact that he had determined to his own satisfaction that many cases of so-called infantile paralysis are due absolutely to peripheral irritation, either septic or physical about the teeth.

I since have been in correspondence with him, and he now has shown me written statements from physicians who have rendered diagnosis of infantile paralysis in cases which have subsequently been cured by treatment to remove the peripheral dental irritation. We know that this disease is regarded as infectious, that it has been quarantined against, that it has been artificially reproduced in animals in one way and another, and yet, here is this other fact of dental peripheral irritation to be taken into account and explained in connection with it.

In the course of my lantern slide presentations, I asked you to consider three cases, one of chorea, one of epilepsy, and another a case of paralysis, all of which were developed from impacted third molars. What does it mean? The type and presumably the intensity of the irritation was the same, the nervous mechanism is presumably the same in all cases, and yet those three different types of reaction were manifested in response to what seems to be the same kind of irritation. There is undoubtedly something in the structure or composition of different individuals that makes one respond one way, and another respond a different way to the same type or degree of irritation. We are testing these people out in various ways as to their physiological and chemical makeup, to see if we can find out why one individual has epilepsy, another chorea, and still another some form of motor paralysis from the same kind of dental irritation. All three cases apparently cured after removal of the irritation. Therein lies the problem.—Dr. E. C. Kirk in *Dental Summary.*

*Think.* A man interested in the Operative and Prosthetic sides of dentistry should constantly bear in mind that every case presents some new phase to the operator, every case is individual. While certain principles underlie all operations, new procedure is called for in each case in just so far as it departs from the typical. This fact injects into the art a constant element of invention. Every case is, to an extent, original. Every case requires some original treatment. This being true, it follows that the progressive dentist will cultivate the fascinating art of invention. He may do this in an unconscious way, nevertheless he cultivates his constructive imagination and thereby constantly strengthens it. No man can achieve a full measure of success without imagination. This faculty, divinely given, is as necessary in professional and business life as it is in poetry and art, and it is all too often neglected upon the specious plea that it is not "practical."

The most practical man is the man who is most useful to himself and the world. He is greatest of value who has the most comprehensive view of life and its problems.

Imagination can be stifled or cultivated like any other faculty. To accept old practice constantly without question, to follow others without understanding, to give up in face of new problems without effort to solve them; these practices stifle and benumb. Question without bombast or irreverence, but question all procedure, consider new methods and meditate upon them, set yourself new problems and study to find their solutions. To do these things is to strengthen and build up your constructive imagination. Out of the ranks of such as do these things come the minds which overthrow error and establish progress.—Dr. C. C. Allen in *Western Journal.*

*Heater for Water and Spray Bottles.* Get an aluminum pan about three inches deep and six to seven inches in diameter (with straight sides if possible). Cut a hole in the side large enough for an electric light receptacle to go through, cut holes in bottom of pan for your glass of water and spray bottles. Put a four-candle power carbon lamp in the receptacle and turn on the juice, and you will have warm water all day long without further bother.—Edward T. Evans, in *Review.*

*Dental Disease and Diet.* Exclusive of dental caries, digestive diseases have increased 103 per cent. within the past thirty years. It would be illuminating to know what the percentage increase would have been had dental disease been included.

All digestive diseases, including dental lesions, are to be ultimately controlled through sane and scientific diet.

People are nowadays said to "dig their graves with their teeth." The sooner people are educated to "eat their way back to health" the better.—Editorial, *Oral Health.*

*Responsible for Work of Assistants.* Better be careful that your assistant doesn't overstep his limits. A Newark, N. J., dentist has just been mulcted in $3,000 damages, affirmed on appeal, because his assistant broke a boy's jaw in extracting, during the absence of the dentist. Court acted under the old principle that employers, as principals, are responsible for the acts of their agents, their employes.—*Summary.*

*Emetin and Heart Failure.* In view of several cases recently recorded in the *British Medical Journal* it is well that enthusiasts in the emetin treatment of pyorrhea should be on their guard against heart failure arising from the use of emetin. It is pointed out that emetin is a powerful agent, whose dosage and the length of time during which it should be administered have to be considered carefully. The depressing effects of the drug, if given in 1-gr. doses daily for some time, becomes very marked, the noticeable features being general lassitude, lowness of spirits, disinclination to make an effort, rapidity of pulse, loss of appetite, nausea, and in some cases difficulty in swallowing and a feeling of constriction about the throat and chest. The question of idiosyncrasy and the fact that symptoms of collapse appeared in the cases recorded three or four days after the drug had been discontinued had to be considered. There could be little doubt that emetin, administered continuously, depressed the heart, for the signs of cardiac depression improved quickly when the drug was omitted. Allan found that a 4-gr. dose caused nausea. Baermann and Heensmain found that 2 to 2½-gr. doses repeated led to weariness and loss of appetite, and that an intermission removed those symptoms.

Ninety per cent. of the dentists in Russia are said to be women. The chief school is the dental college at Petrograd which has six hundred students, less than five per cent. of whom are of the sterner sex. Warsaw, Moscow, Odessa, and Kiev, also boast of large dental schools. The course is three years and the fees for tuition amount to approximately $100 per year. The State diploma costs $10 and a license to practice is granted only after proof of qualification. The first year is devoted to mechanical dentistry and the subsequent years to clinical work from ten A. M. to five P. M. After this follow lectures until nine P. M. Examinations are held every month. The lecturers at Petrograd number about twelve and come from the Imperial

University Faculty of Medicine. The ten demonstrators are chiefly women. Fees in both medicine and dentistry are considerably lower than in America and hours of work are longer. Men seem to prefer the medical profession, and it is said that even in Petrograd the men dentists of standing do not number more than half a dozen.—*Oral Hygiene.*

*A Handy Matrix for Large Filling Restorations.* One of the most satisfactory matrices for use in those cases where it is necessary to embrace the whole tooth, especially where the cavity extends below the gingival margin to any extent, is the ordinary anchor clamp band used for orthodontia work. After removing the tube for carrying the bar the band can be quickly and easily adjusted to the tooth and screwed up. It is a much more satisfactory method than the usual hand-made matrix, and has the double advantage of being able to be used again and again for the purpose.—W. Stanley Wilkinson in *Summary.*

*Glycerine.* Glycerine has long been regarded as possessing some degree of antiseptic power but it has been proven to be a most efficient sterilizing agent. It is particularly suited for steel instruments, making them absolutely aseptic without injury to temper or surface. The glycerine is heated to a temperature of 120 degrees centigrade and steel instruments can remain in the solution for an hour or more and no injury. A minute is said to kill all germs, and rubber tubes are not only uninjured, but restored to their original elasticity.—*Oral Hygiene.*

*An Experience with Mercuric Succinimid.* The following experience may interest those who may contemplate using mercuric succinimid in the treatment of pyorrhea. Mrs. B. presented with pyorrhea of the lower teeth. Owing to her modesty the injection of 3-5 grain of mercuric succinimid was made in the deltoid muscle.

About fourteen hours later, at 2 A. M., she awakened with very violent abdominal pains and pain all over the body. Her family physician was called and not knowing the cause of her condition he could not proceed intelligently with the case. She was confined to her bed for some days, and a month later a large inflamed area similar to a vaccination mark existed at the point of injection.

Aseptic precaution was observed at the time of injection, so the reaction was no doubt caused by the drug. Needless to say, the patient did not continue with the mercuric treatment.—George E. Cox, D.D.S., in August *Cosmos.*

*To Relieve Pain in Incipient Alveolar Abscess or in Neuralgia.* Sometimes the pain is vague along one side of the jaw and not easily located. If it is found impossible to decide on any one tooth as the seat of the trouble, the pain may frequently be relieved by taking some menthol crystals, about the quantity that would lie on a dime, and placing them in half a teaspoon of water and holding the spoon over the gas till the crystals are melted down in the liquid. Dip some cotton in the melted menthol and pack this between the cheek and gum on the affected side. The menthol will sooth the pain and frequently relieve it entirely. This is also an excellent remedy for clearing out the head and facilitating the breathing where the nasal passages are stopped up with a cold. In this case pack the saturated cotton under the upper lip. It will be found a great relief. —*Dental Review.*

